# Spanish translation and cross-cultural adaptation and validation of the social motivational orientations in sport scale for children

**DOI:** 10.3389/fspor.2026.1743553

**Published:** 2026-02-04

**Authors:** Raquel Pastor-Cisneros, María Mendoza-Muñoz, José Francisco López-Gil, Jorge Carlos-Vivas

**Affiliations:** 1 Physical Activity for Education, Performance and Health (PAEPH) Research Group, Faculty of Sport Sciences, University of Extremadura, Cáceres, Spain; 2 Department of Communication and Education, Universidad Loyola Andalucía, Sevilla, Spain; 3 School of Medicine, Universidad Espíritu Santo, Samborondón, Ecuador; 4 Vicerrectoría de Investigación y Postgrado, Universidad de Los Lagos, Osorno, Chile

**Keywords:** Assessment, exercise, physical activity, physical literacy, school children, SPLA-C

## Abstract

**Background:**

Social motivation plays a key role in sports participation but is underexplored in measurement tools. The Social Motivational Orientations in Sport Scale (SMOSS) is the only instrument assessing these orientations; however, no Spanish version exists for children. The primary objective of this study was to culturally adapt and psychometrically validate a Spanish version of the SMOSS for children aged 6–12 years.

**Methods:**

The SMOSS for children (SMOSS-C) was translated and culturally adapted. Comprehension was evaluated through cognitive interviews, and reliability was assessed via test–retest with 128 Spanish schoolchildren. Analyses included confirmatory factor analysis (CFA), Cronbach's alpha (*α*), and intraclass correlation coefficient (ICC).

**Results:**

CFA confirmed the three-factor structure (affiliation, recognition, status) with excellent fit indices (*χ*^2^/df = 1.667, RMSEA =.072, CFI =.943, TLI =.926). Internal consistency ranged from questionable to good (*α* = .636–.891), except for items 6 and 15 (poor/unacceptable). Temporal stability was moderate to near-perfect (ICC =.467–.891), with items 6 and 15 showing only fair agreement. Measurement error was low for the total score (SEM% = 7.0; MDC% = 19.5), indicating good accuracy.

**Conclusions:**

The SMOSS-C shows a valid factorial structure, acceptable internal consistency, and good temporal reliability for most items and the overall score. Despite two weaker items, it is a reliable tool for assessing social motivational orientations in sport among Spanish schoolchildren. It enhances understanding of social influences on motivation for physical activity and informs pedagogical strategies. As part of the social domain, the SMOSS-C supports the Spanish Physical Literacy Assessment for Children (SPLA-C) model, the first physical literacy assessment instrument in Spain.

## 1 Introduction

Human motivation is a complex and multifaceted phenomenon resulting from the interaction of intrinsic and extrinsic factors, with social factors playing a pivotal role (1). Achievement motivation theory (2) analyses how various needs, such as the desire for achievement, power and affiliation, influence individual motivation in different contexts. In this context, Nicholls and Maehr (2) identified social approval as one of the primary social objectives within the three broad, universal motivations. This perspective has prompted research providing empirical evidence of the influence of social approval in the sporting context (3, 4).

Motivation has been identified as a key factor in sport, as it directly affects athletes' performance, persistence and satisfaction (5). Motivation can manifest both intrinsically, on the basis of enjoyment, interest and self-improvement, and extrinsically, on the basis of external rewards and recognition (6). In this context, social motivation is particularly relevant, as the need for approval and recognition from peers, coaches or spectators can strengthen commitment to sport and encourage group cohesion in team sports (7–9). Moreover, it can also impact self-image and performance in individual disciplines.

Several instruments have been developed to assess motivation in sports, primarily on the basis of theoretical frameworks such as self-determination theory (10) and achievement goal theory (2). Popular questionnaires such as the Sport Motivation Scale (SMS) (11) and the Perception Of Success Questionnaire (POSQ) (12) have enabled the exploration of intrinsic and extrinsic motivational factors, as well as task or ego orientation. However, social motivation has received less attention in terms of measurement. Instruments such as the Social Motivation in Sport Scale (SMOSS) (13) address this dimension more directly and provide a useful means of investigating the impact of social factors on participation in sport. This is particularly relevant in educational contexts, where social factors such as affiliation, status and recognition can be important motivators of participation and engagement in both structured sports and physical education (PE) (14, 15).

Although the SMOSS was introduced two decades ago, it remains the only instrument available for measuring social motivational orientations in sports. While some studies have adapted the tool for use in PE (16, 17), to our knowledge, only three studies (18–20) have evaluated its psychometric properties in adolescents. Furthermore, to date, the SMOSS has only been translated and adapted into Portuguese (18), and there is still no validated version for the Spanish population. However, given that our target population is children aged 6–12, no adaptations for this age group have yet been developed, representing a significant gap in the literature. It is therefore essential to adapt the original instrument rather than apply it directly, considering sociocultural and contextual differences (21, 22), as well as the cognitive and linguistic variations (23) between children and adolescents/adults. This will enable a more accurate evaluation of the social motivations that influence children's participation in sports.

Therefore, the main objective of the present study was to validate a Spanish version of the SMOSS adapted specifically for use with Spanish schoolchildren aged 6–12 years, resulting in the SMOSS for children (SMOSS-C). This will allow for a deeper understanding of, and evaluation of, the social factors influencing participation in PE classes and will facilitate the design of intervention programs that effectively address the difficulties encountered in this area.

## 2 Material and methods

### 2.1 Study design

This instrumental, cross-sectional methodological study aimed to linguistically and culturally adapt the Social Motivational Orientations in Sport Scale (SMOSS), originally designed for adolescents and young adults, for use with Spanish children aged 6–12 years. The adapted version (SMOSS-C) targets the developmental characteristics of primary education students.

The study was carried out in three main phases:

Linguistic and cultural adaptation:

The vocabulary, sentence structure, and content of the original SMOSS were adjusted to align with the cognitive, linguistic, and socioemotional development of children in the 6–12 age group. This adaptation process included direct and reverse translation of the original items, following the World Health Organization (WHO) recommendations for adapting instruments (24). After translation, three experts in children's physical literacy, education, and motivation in physical activity reviewed each item to ensure developmental appropriateness.

## 3 Assessment of comprehension through cognitive interviews

To evaluate the clarity and functional understanding of the adapted items, individual cognitive interviews were conducted with a pilot sample of schoolchildren (*n* = 17). Cognitive interviews were conducted with 17 children (4 in 2nd grade, 4 in 3rd, 3 in 4th, 3 in 5th, and 3 in 6th), following WHO guideline steps and expert panel review; 3 of the initially invited 20 children missed one of the sessions. These interviews focused on analyzing both literal and contextual comprehension. Understanding was also evaluated through an interview process aligned with best practices to ensure child-friendly instruments (25).

## 4 Psychometric analysis and test-retest reliability

In the second phase, the psychometric properties of the SMOSS-C were analyzed. This included a confirmatory factor analysis (CFA) to assess construct validity. Additionally, a test‒retest reliability study was carried out with a subsample of children who completed the questionnaire twice, with a two-week interval, to assess the temporal stability of the responses.

This rigorous, phased approach ensured that the SMOSS-C was not only linguistically and culturally appropriate for Spanish children but also psychometrically sound in terms of content validity, functional comprehension, and reliability.

The final version of the adapted instrument is available in Supplementary Material S1.

### 4.1 Participants

A total of 128 schoolchildren (63 boys and 65 girls), aged between 6 and 12 years [9.57 (±1.80) years] were recruited from different primary schools in Extremadura, Spain. The participants were selected via nonprobability convenience sampling. The inclusion criteria were (a) being enrolled in primary education; (b) having provided informed consent signed by a parent or legal guardian; and (c) having an adequate level of reading comprehension to complete the adapted questionnaire.

### 4.2 Ethical approval

This study involved human participants and was approved by the Bioethics and Biosafety Committee of the University of Extremadura (approval number: 288/2024). The updates of the Declaration of Helsinki, amended by the 75th General Assembly of the World Medical Association (Helsinki 2024) and Law 14/2007 on Biomedical Research, were followed. The participants provided informed consent to participate in the study before taking part**.**

### 4.3 Instrument and translation-adaptation process

The data were collected via an adapted version of the original SMOSS [15], which is designed to assess social motivations in the context of sport and is based on achievement motivation theory 2. The questionnaire comprises 15 items distributed across three subscales: affiliation, recognition and status. Responses were collected via a 5-point Likert scale ranging from 1 (“Strongly disagree”) to 5 (“Strongly agree”). To adapt the instrument for use with children, the SMOSS items underwent a process of translation and linguistic and cultural adaptation (see Table 1). These adaptations consisted of a) translation and back-translation; b) simplifying vocabulary and syntax; and c) eliminating or replacing abstract or difficult-to-interpret concepts. Version 1 of the questionnaire included words, expressions and concepts modified on the basis of the consensus of two previous translations, as detailed in Table 1. Version 1 was then retranslated by a native English speaker with good knowledge of Spanish. They compared retranslation with the original English version and found no significant differences.

**Table 1:** Different versions of the social motivational orientations in sport scale for children.

Items	English versión (original)	Translation 1	Translation 2	Version 1. An agreed version of the translations	Final version. Adaptations after cognitive interviews
Opening sentence	“I feel things have gone well in my sport when..”	“Siento que las cosas han salido bien en mi deporte cuando…”	“Me siento bien en mi deporte cuando…”	“Me siento bien en mi deporte cuando…”	“Me siento bien en mi deporte cuando…”
1	Others tell me I have performed well	Los demás me dicen que lo he hecho bien	Los demás me dicen que lo hice bien.	Los demás me dicen que lo he hecho bien.	Los demás me dicen que lo he hecho bien.
2	I make some good friends on the team	Hago buenos amigos en el equipo	Hago buenos amigos en el equipo.	Hago buenos amigos en el equipo.	Hago buenos amigos en el equipo.
3	I belong to the popular group in the team	Pertenezco al grupo popular del equipo	Soy parte del grupo popular del equipo.	Soy parte de los populares del equipo.	Soy parte de los populares del equipo.
4	My teammates and I have a laugh together	Mis compañeros de equipo y yo nos reímos juntos	Mis compañeros y yo nos reímos juntos.	Mis compañeros de equipo y yo nos reímos juntos	Mis compañeros de equipo y yo nos reímos juntos
5	I am the center of attention	Soy el centro de atención	Soy el centro de atención.	Soy el centro de atención.	Soy el centro de atención.
6	I make new friends who I socialize with outside sport	Hago nuevos amigos con los que salir fuera del deporte	Hago nuevos amigos con los que juego fuera del deporte.	Hago nuevos amigos con los que me junto fuera del deporte.	Hago nuevos amigos con los que me junto fuera del deporte.
7	I have fun with the others on my team	Me divierto con los demás de mi equipo	Me divierto con los demás de mi equipo.	Me divierto con los demás de mi equipo.	Me divierto con los demás de mi equipo.
8	I am part of the “in” crowd	Soy parte de la gente “de moda”.	Soy parte de los niños “de moda”.	Soy parte de los niños “de moda”.	Soy parte de los niños populares.
9	Other kids think I’m truly good at sport	Otros niños piensan que soy muy bueno en el deporte	Los demás niños piensan que soy muy bueno en el deporte.	Los demás niños piensan que soy muy bueno en el deporte.	Los demás niños piensan que soy muy bueno en el deporte.
10	I receive recognition from others for my accomplishments	Recibo el reconocimiento de los demás por mis logros	Los demás me felicitan por mis logros.	Los demás me felicitan por mis logros.	Los demás me felicitan por mis logros.
11	Spending time with the other players is enjoyable	Disfruto pasando el tiempo con el resto de los jugadores	Pasar tiempo con los otros jugadores es divertido.	Disfruto pasando el tiempo con el resto de los jugadores.	Disfruto pasando el tiempo con el resto de los jugadores.
12	I become friends with some of the others in my sport	Me hago amigo de los demás en mi deporte	Me hago amigo de otros niños que practican mi deporte.	Me hago amigo de otros niños que practican mi deporte.	Me hago amigo de otros niños que practican mi deporte.
13	Others are impressed by my sporting ability	A los demás les impresiona mi habilidad deportiva	A los demás les impresiona lo bien que juego.	A los demás les impresiona lo bien que juego.	A los demás les impresiona lo bien que juego.
14	I am one of the more popular players	Soy uno de los jugadores más populares	Soy uno de los jugadores más populares.	Soy uno de los jugadores más populares.	Soy uno de los jugadores más populares.
15	Just hanging out with the others is fun	Me divierto pasando el rato con los demás.	Pasar el rato con los demás es divertido.	Me divierto pasando el rato con los demás.	Me divierto pasando el rato con los demás.

The adaptation of the SMOSS resulted in the SMOSS-C, which retained the original theoretical dimensions. As the adaptation was based on an established structure, a CFA was performed to verify the validity of the original structure in the new population. A panel of experts, in the field of physical literacy and PE in primary schools, reviewed the content of each item, and cognitive interviews were conducted with a pilot group of 17 children to verify the clarity, comprehension and suitability of each item. Virtually no comprehension problems were identified by the participants, all of whom rated the scale as clear and understandable, except for item 8, which underwent slight modification (see Table 1).

### 4.4 Statistical analyses

All the data gathered were recorded in a database designed for this research project, with personal data remaining anonymous. Statistical analyses were performed via the Statistical Package for the Social Sciences (SPSS, version 25.0; IBM SPSS Inc., Armonk, NY, USA). CFA was performed via the software package AMOS v.23.0.0 (IBM Corporation, Wexford, PA, USA). The different items of the SMOSS (Spanish version) were included as elements. To assess the model's goodness of fit, the following indices were selected: (1) the chi-square probability with appropriate nonsignificant values (*p* > 0.05) (26), (2) the root mean square error of approximation (RMSEA) (27), (3) the comparative fit index (CFI), (4) the Tucker‒Lewis index (TLI), and (5) the chi-square per degree of freedom ratio (CMIN/DF) (28).

Item-level analysis included the calculation of means, standard deviations, and response distributions. To evaluate the sensitivity of the scale, floor and ceiling effects were determined based on the percentage of participants achieving the minimum and maximum possible scores, respectively. Univariate normality was assessed through skewness and kurtosis indices. Regarding missing data, the dataset was screened for completeness; as no missing observations were identified (0% missingness rate), no imputation procedures were required. Additionally, comprehensibility scores for each item were analyzed and compared across age subgroups (6–7, 8–9, and 10–12 years).

Internal consistency and reliability were evaluated via Cronbach's alpha and McDonald's omega coefficients. The inclusion of omega (calculated via Maximum Likelihood estimation) provided a more robust reliability estimate by addressing the limitations of alpha regarding the assumption of tau-equivalence, which is particularly relevant in child samples. According to Glen (29), Cronbach's *α* can be interpreted as follows: 0.5 signifies unacceptable; 0.5–0.6 indicates poor; 0.6–0.7 suggests questionable; 0.7–0.8 is acceptable; 0.8–0.9 is good; and 0.9 represents excellent. Item-total correlations and discrimination analyses were also performed to ensure item-level validity.

A test–retest reliability check was performed 15 days later. Stability was evaluated by computing the intraclass correlation coefficient (ICC) with a 95% confidence interval using a two-way random effects model (absolute agreement) (30). ICC scores were interpreted on the basis of the standards provided by Landis et al. (31): <0.20 indicates slight agreement; 0.21–0.40, fair; 0.41–0.60, moderate; 0.61–0.80, substantial; and 0.80, near perfection. Additionally, the standard error of measurement (SEM) and the minimum detectable change (MDC) (32) were employed to evaluate absolute reliability. Spearman's rho (*ρ*) correlation was performed to analyze the relationship between each item and the total score. The significance level was set at *p* < 0.05 for all tests. Finally, to further assess agreement and identify potential systematic bias between measurement points, a Bland–Altman plot was generated, including the mean difference (bias) and the 95% limits of agreement (±1.96 × SD). The significance level was set at *p* < 0.05 for all tests.

## 5 Results

### 5.1 Item analysis and descriptive statistics

Descriptive statistics for the 15 items of the SMOSS-C questionnaire at both test and retest are presented in Table 2. Regarding missing data, the dataset was complete with a 0% missingness rate across all items (*N* = 128 valid observations), confirming the feasibility of the administration process. Consequently, no imputation methods were required.

**Table 2:** Item distributions, ceiling/floor effect percentages, and comprehensibility scores of the SMOSS-C questionnaire by age subgroups for test and retest.

Items	M	SD	N valid	N missing; %	N floor; %	N ceiling; %	Skewness	Kurtosis	Comprehensibility 6–7	Comprehensibility 8–9	Comprehensibility 10–12
Test											
Item 1	4.27	1.18	128	0; 0	8; 6.3	81; 63.3	−1.612	1.598	4.32	4.09	4.30
Item 2	4.37	1.11	128	0; 0	6; 4.7	87; 68.0	−1.796	2.332	4.65	4.13	4.32
Item 3	3.13	1.50	128	0; 0	30; 23.4	32; 25.0	−0.188	−1.351	3.03	3.65	3.00
Item 4	4.25	1.20	128	0; 0	7; 5.5	82; 64.1	−1.514	1.114	4.29	4.17	4.26
Item 5	2.48	1.46	128	0; 0	49; 38.3	18; 14.1	0.475	−1.180	3.26	2.61	2.12
Item 6	3.97	1.42	128	0; 0	15; 11.7	73; 57.0	−1.089	−0.279	4.26	3.57	3.97
Item 7	4.47	0.90	128	0; 0	2; 1.6	85; 66.4	−1.875	3.308	4.55	4.43	4.45
Item 8	3.05	1.56	128	0; 0	32; 25.0	35; 27.3	−0.041	−1.509	3.42	3.57	2.73
Item 9	3.70	1.38	128	0; 0	15; 11.7	51; 39.8	−0.737	−0.700	3.65	3.97	3.64
Item 10	4.05	1.21	128	0; 0	7; 5.5	64; 50.0	−1.150	0.275	4.06	4.13	4.01
Item 11	4.30	1.05	128	0; 0	4; 3.1	77; 60.2	−1.506	1.625	4.52	4.17	4.24
Item 12	4.20	1.15	128	0; 0	7; 5.5	73; 57.0	−1.431	1.220	4.32	4.09	4.18
Item 13	3.39	1.32	128	0; 0	14; 10.9	35; 27.3	−0.319	−1.007	3.32	3.48	3.39
Item 14	2.86	1.47	128	0; 0	35; 27.3	25; 19.5	0.096	−1.319	2.97	3.30	2.68
Item 15	4.48	1.00	128	0; 0	5; 3.9	90; 70.3	−2.264	4.658	4.39	4.39	4.55
Retest											
Item 1	3.92	1.43	128	0; 0	15; 11.7	69; 53.9	−1.029	−0.407	3.90	3.61	4.03
Item 2	4.36	0.95	128	0; 0	2; 1.6	78; 60.9	−1.499	1.700	4.58	4.09	4.35
Item 3	3.01	1.54	128	0; 0	34; 26.6	31; 24.2	−0.052	−1.501	2.42	3.70	3.04
Item 4	4.18	1.23	128	0; 0	9; 7.0	76; 59.4	−1.444	1.010	4.19	3.78	4.30
Item 5	2.40	1.41	128	0; 0	50; 39.1	16; 12.5	0.578	−0.958	2.45	2.83	2.24
Item 6	4.01	1.30	128	0; 0	10; 7.8	68; 53.1	−1.097	0.010	4.10	4.00	3.97
Item 7	4.38	0.99	128	0; 0	3; 2.3	82; 64.1	−1.677	2.255	4.55	4.17	4.38
Item 8	2.85	1.52	128	0; 0	37; 28.9	28; 21.9	0.132	−1.403	2.65	3.48	2.74
Item 9	3.77	1.24	128	0; 0	10; 7.8	48; 37.5	−0.754	−0.322	3.97	4.13	3.57
Item 10	4.09	1.15	128	0; 0	6; 4.7	62; 48.4	−1.269	0.757	4.19	4.17	4.03
Item 11	4.38	1.07	128	0; 0	7; 5.5	83; 64.8	−2.021	3.475	4.55	4.30	4.34
Item 12	4.22	1.18	128	0; 0	7; 5.5	77; 60.2	−1.466	1.149	4.19	4.30	4.20
Item 13	3.39	1.28	128	0; 0	15; 11.7	29; 22.7	−0.446	−0.753	3.26	3.70	3.35
Item 14	2.83	1.52	128	0; 0	38; 29.7	26; 20.3	0.132	−1.451	2.65	3.39	2.73
Item 15	4.50	1.06	128	0; 0	6; 4.7	97; 75.8	−2.266	4.171	4.35	4.52	4.55

Item-level analysis indicated a variety of response distributions. Skewness values ranged from −2.264 to 0.578, and kurtosis values ranged from −1.509 to 4.658. Several items (e.g., Items 1, 2, 7, 11, and 15) exhibited negative skewness exceeding the 1.0 threshold, which is consistent with the high mean scores observed (>4.0). Specifically, Item 15 showed the highest kurtosis (4.658), suggesting a leptokurtic distribution. These departures from multivariate normality justify the use of robust estimators in subsequent Factor Analyses.

Significant ceiling effects (defined as >15% of participants achieving the maximum score) were observed in most items, particularly Items 1, 2, 4, 6, 7, 10, 11, 12, and 15, with Item 15 reaching the highest ceiling effect at 75.8% during retest. Conversely, floor effects (>15%) were notably present in Items 3 (23.4%), 5 (38.3%), 8 (25.0%), and 14 (27.3%). These results suggest that while the instrument effectively captures the intended constructs, some items may have limited sensitivity at the upper or lower ends of the scale in this specific population.

Comprehensibility scores remained high across all developmental stages, with mean values generally exceeding 3.0 on a 5-point scale. Subgroup analysis (6–7, 8–9, and 10–12 years) showed that the oldest group (10–12 years) generally reported the highest levels of clarity. However, Items 5 and 14 showed slightly lower comprehensibility scores in the youngest and oldest groups compared to the middle group, although they remained within acceptable ranges for instrument utility.

### 5.2 Confirmatory factor analysis (CFA)

**Figure 1:**
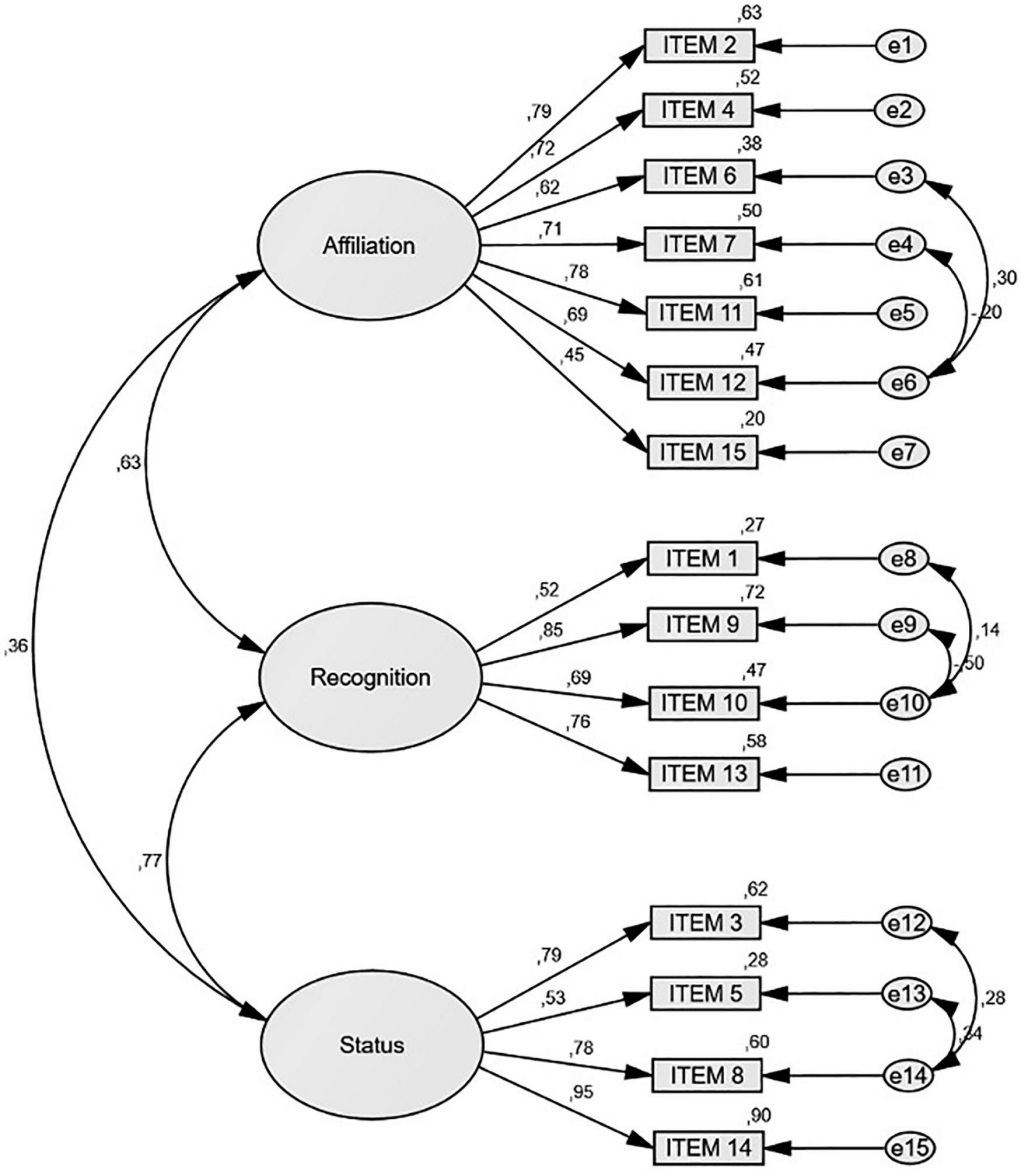
Overview of research to develop the SMOSS-C.

CFA was conducted with a total of 128 participants aged 9.57 (±1.80) years, of whom 49.2% were boys and 50.8% were girls. Figure 1 illustrates the resulting model from the CFA.

A good model fit was shown by the confirmatory factor analysis (Table 3). The acceptable range was reached (CMIN/df = 1.667) (33). Although the chi-square test was statistically significant (*p* < .001), this is common in large samples and does not necessarily indicate poor fit (34). The CFI was .943, and the TLI was .926, both approaching the recommended threshold of.95 for excellent fit and exceeding the .90 cutoff for acceptable fit (35). The RMSEA was .072, indicating a reasonable error of approximation, as values below .080 are generally considered acceptable (36). Overall, these results support the adequacy of the proposed factorial structure.

**Table 3:** Social motivational orientations in sport scale for children goodness-of-fit indices.

Indices	Value
CMIN/DF	1.667
*p* (*χ*^2^)	<.001
RMSEA	.072
CFI	.943
TLI	.926

CMIN/DF, minimum discrepancy per degree of freedom; *p* (χ^2^), chi-square probability; RMSEA, root mean square error of approximation; CFI, comparative fit index; TLI, Tucker‒Lewis index.

### 5.3 Test-retest reliability and internal consistency

Table 4 displays the internal consistency, reproducibility and systematic differences of the SMOSS-C. Overall, the internal consistency ranged from questionable to good for all the items and the total score of the questionnaire (Cronbach's *α* from .636 to .891), except for items 6 (Cronbach's *α* = .531) and 15 (Cronbach's *α* = .466), where it was poor and unacceptable, respectively. All items at the initial and follow-up tests significantly correlated with total SMOSS-C scores [Spearman's rho (*ρ*): .337 to .777].

**Table 4:** Reliability, test‒retest, and systematic differences in the SMOSS-C score.

Items	Test (*n* = 128)			Retest (*n* = 128)			Reliability test						
	M	SD	Item-Total Correlation	M	SD	Item-Total Correlation	Cronbach's *α*	ICC (95% CI)	*p* value^†^	SEM	%SEM	MDC	%MDC
Item 1	4.27	1.18	.516**	3.92	1.43	.674**	.756	.590 (.453 to.697)	.001	0.84	20.4	2.32	56.6
Item 2	4.37	1.11	.573**	4.36	0.95	.724**	.694	.534 (.397 to.647)	.929	0.70	16.1	1.95	44.6
Item 3	3.13	1.50	.724**	3.01	1.54	.721**	.825	.701 (.601 to.779)	.260	0.83	27.1	2.30	75.0
Item 4	4.25	1.20	.571**	4.18	1.23	.559**	.640	.472 (.325 to.596)	.523	0.88	20.9	2.45	58.1
Item 5	2.48	1.46	.579**	2.40	1.41	.677**	.636	.467 (.320 to.592)	.512	1.05	42.9	2.90	119.0
Item 6	3.97	1.42	.546**	4.01	1.30	.588**	.531	.363 (.202 to.504)	.773	1.09	27.2	3.01	75.4
Item 7	4.47	0.90	.564**	4.38	0.99	.627**	.798	.663 (.553 to.749)	.210	0.55	12.4	1.52	34.4
Item 8	3.05	1.56	.644**	2.85	1.52	.661**	.773	.626 (.509 to.721)	.097	0.94	31.9	2.61	88.5
Item 9	3.70	1.38	.753**	3.77	1.24	.723**	.702	.542 (.407 to.653)	.526	0.89	23.7	2.46	65.8
Item 10	4.05	1.21	.648**	4.09	1.15	.638**	.770	.628 (.510 to.722)	.602	0.72	17.7	1.99	49.0
Item 11	4.30	1.05	.577**	4.38	1.07	.485**	.710	.551 (.418 to.661)	.333	0.71	16.4	1.97	45.4
Item 12	4.20	1.15	.573**	4.22	1.18	.610**	.765	.621 (.502 to.717)	.793	0.72	17.0	1.99	47.2
Item 13	3.39	1.32	.721**	3.39	1.28	.689**	.778	.638 (.523 to.731)	>.999	0.78	23.1	2.17	64.0
Item 14	2.86	1.47	.777**	2.83	1.52	.688**	.812	.685 (.580 to.767)	.766	0.84	29.5	2.33	81.7
Item 15	4.48	1.00	.337**	4.50	1.06	.445**	.466	.305 (.139 to.455)	.884	0.86	19.1	2.38	53.0
Total SMOSS-C score	56.95	11.91	N/A	56.29	12.16	N/A	.891	.891 (.845 to.923)	.325	3.97	7.0	11.01	19.5

M, mean; SD, standard deviation; 95% CI, confidence interval of 95%; ICC, intraclass correlation coefficient; SEM, standard error of measurement; %SEM, standard error of measurement percentage; MDC, minimum detectable change; %MDC, minimum detectable change percentage; N/A, not applicable; SMOSS-C, social motivational orientations in sport scale for children. ^†^Friedman test *p* values. The item-total correlation refers to the magnitude of the association between each item and its domain.

** Significant correlation at *p* < 0.01.

The reproducibility outcomes revealed moderate to near perfection test–retest reliability for each item and the total SMOSS-C score (ICC: .467 to .891), except for items 6 (ICC = .363) and 15 (ICC = .305), which showed fair agreement. The SEM and %SEM values for each item and the total SMOSS score ranged from 0.55–3.97 and from 7.0-42.9, respectively. Similarly, the MDC and %MDC values for each item and the total SMOSS score ranged from 1.52-11.01 and from 19.5-119.0, respectively. Moreover, comparison outcomes overall showed no significant differences for any of the items or the total SMOSS-C score (*p* > .05), except for item 1 (*p* = .001).

Additionally, McDonald's omega was also calculated to provide a more robust estimation of reliability. For the total scale, McDonald's omega was .892 at test and .886 at retest. These values, being well above the recommended threshold of .70, indicate high internal consistency and suggest that the instrument maintains strong reliability across different time points. Furthermore, the similarity between the test and retest omega values confirms the structural stability of the SMOSS-C over time.

**Figure 2:**
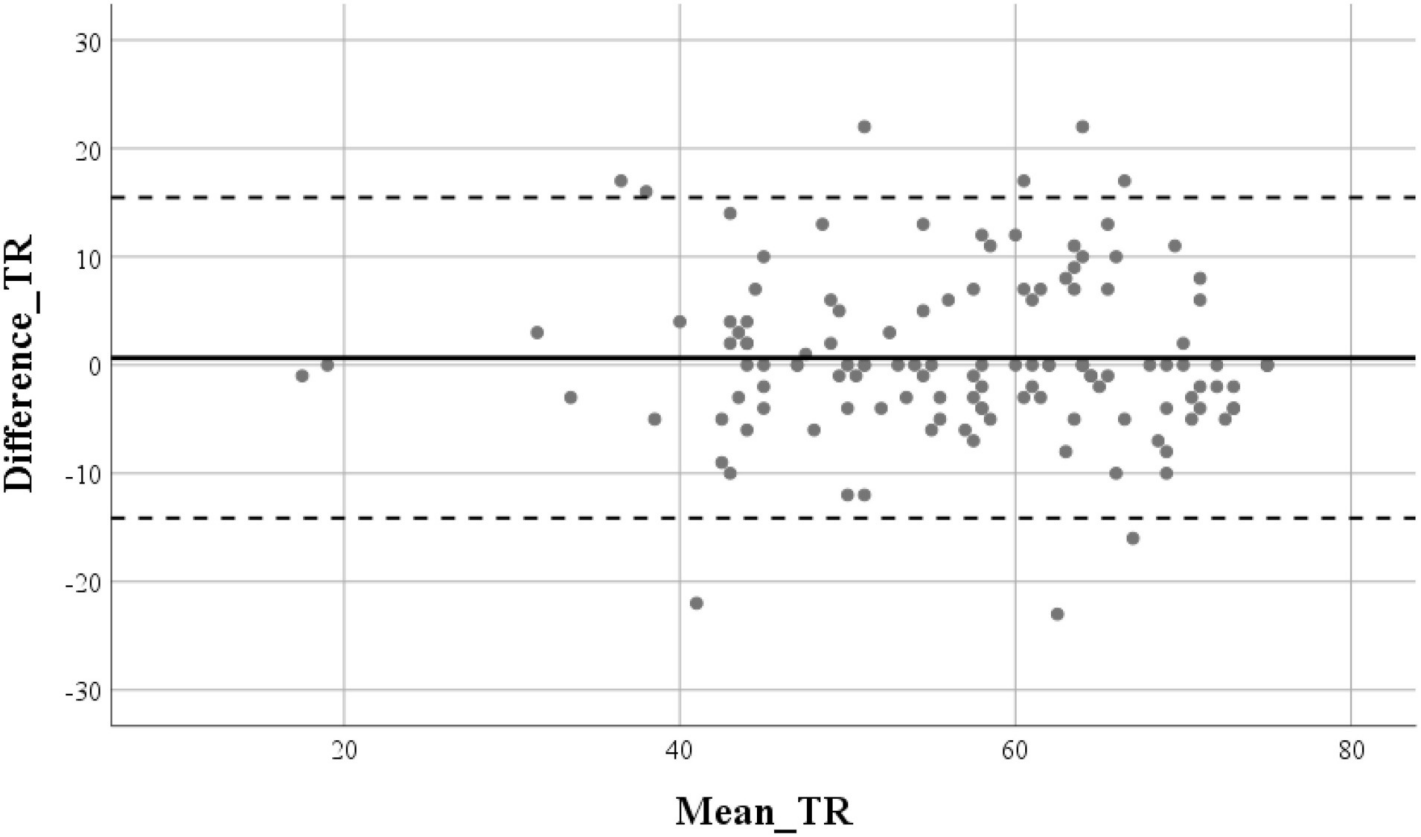
Bland-Altman plot for test-retest reliability of the SMOSS-C total scores (*n* = 128). The *Y*-axis represents the difference between the test and retest scores, and the *X*-axis represents the mean of the two measurements. The solid horizontal line indicates the mean difference (bias), while the dashed lines represent the 95% limits of agreement (±1.96 × SD).

Finally, the temporal stability of the SMOSS-C was further evaluated using a Bland-Altman plot to assess the agreement between test and retest total scores (*n* = 128). As shown in Figure 2, the mean difference (bias) between the two measurement points was 0.66, indicating no significant systematic overestimation or underestimation of the scores over time. The 95% limits of agreement ranged from −14.14 to 15.46. Visual inspection of the plot reveals that the vast majority of data points (>95%) fell within these limits, confirming a high degree of consistency at the individual level. Furthermore, the distribution of differences remained stable across the range of mean scores, suggesting the absence of proportional bias. These findings, in conjunction with the internal consistency metrics, provide robust evidence for the reliability and stability of the SMOSS-C in the target population.

## 6 Discussion

This study aimed to adapt the SMOSS, which was originally designed for adolescents and young adults, for its use with Spanish children aged 6–12 years (SMOSS-C). The study also analyzed the psychometric properties of the adapted instrument, with a focus on its factor structure, internal consistency and temporal stability. Our findings support the validity and reliability of the SMOSS-C for its use with this age group. The CFA showed a good fit between the model and the data, suggesting that the questionnaire's theoretical structure is appropriate for this age group. Reliability analyses revealed that the instrument has acceptable to excellent internal consistency for most items, as well as high temporal stability for the total score. However, items 6 and 15 were identified as having low consistency and test‒retest reliability values, suggesting that they may not function adequately in this population and may require revision and even removal in future versions of the questionnaire. Overall, these findings indicate that the SMOSS-C is a valuable and promising instrument for evaluating the social motivational orientations of school-aged children, establishing a robust foundation for its application in educational and research settings.

The CFA of the SMOSS-C revealed that the theoretical model fit the empirical data adequately, thus supporting the structural validity of the questionnaire in the child population. Specifically, the CMIN/df value (1.667) was within the range considered to indicate an excellent fit (<2), and the CFI (.943) and TLI (.926) indices far exceeded the threshold of .90, approaching the (35) and Bentler (35) criterion of excellence (.95). Additionally, the RMSEA value (.072) remained within the acceptable range (<0.08), indicating a reasonable approximation error. While the chi-square test was statistically significant (*p* < .001), this is a common occurrence in moderate or large samples and should not be interpreted as evidence of a poor fit in isolation (34). Therefore, taken together, the obtained indices suggest that the instrument's original factor structure remains adequate after its linguistic and conceptual adaptation for use with Spanish schoolchildren. These results are particularly relevant given that the SMOSS-C was applied to children aged 6–12, who are at a stage of development with specific cognitive and linguistic characteristics (37). The adequacy of the factorial model indicates that children consistently understand and respond to the various items assessing social motivational orientations in a sporting context. This further supports the value of the questionnaire as a valid tool for use in primary education.

Regarding the internal consistency outcomes of the SMOSS-C, the total score showed excellent consistency (Cronbach's *α* = .891), indicating high homogeneity among the items and their ability to consistently measure the construct of social motivational orientation in sport, on the basis of achievement motivation theory (2). At the item level, most items had *α* values ranging from acceptable to good, which reinforced the internal consistency of the instrument. However, it is important to note that items 6 (Cronbach's Cronbach's *α* = .531) and 15 (Cronbach's *α* = .466) showed poor and unacceptable levels of consistency, respectively. These results suggest that these items may not adequately capture the construct in this child population, possibly because of factors such as language complexity, ambiguous wording or difficulty interpreting them (38). Reformulating these problematic items could improve the instrument's overall reliability, particularly for its use in longitudinal studies. Overall, the internal consistency of the SMOSS-C is solid, although there is room for improvement in certain items.

The test‒retest reliability assessment of the SMOSS-C revealed positive results in terms of the instrument's temporal stability. The total questionnaire score obtained an ICC of .891, indicating an excellent degree of agreement between measurements taken 15 days apart. This finding supports the robustness of the instrument for reliably assessing social motivational orientations in children's sports contexts. At the item level, most items had ICC values ranging from moderate to nearly perfect, suggesting that the children responded consistently over time. However, as in the internal consistency analysis, specific problems were detected in items 6 (ICC = .363) and 15 (ICC = .305), which showed low levels of stability and achieved only “fair” agreement according to the criteria of Landis and Koch (31). This reinforces the need to revise these items, as their low stability could be due to variable interpretations or comprehension difficulties on the part of the children's group. These results are consistent with those of most motivation questionnaires for children, which demonstrate good or excellent test‒retest reliability, with ICCs typically ranging from moderate to high (39–41). However, some subscales or items tend to demonstrate lower reliability (42, 43), and cultural adaptation, the use of abstract language, or social factors may affect the outcomes (44). Similarly, research on physical literacy questionnaires, such as the CAPL-2 (45), has identified issues with the stability of items when applied to younger groups. This suggests that this is a common challenge in the field of child psychometric assessment.

Similarly, absolute error analyses (SEM and MDC) indicated that the measurement error was low in the total score (%SEM = 7.0; %MDC = 19.5), further supporting the overall accuracy of the instrument. However, some individual items presented high %SEM and %MDC values (e.g., item 5 with an %SEM of 42.9), which could reflect greater variability in children's responses and suggest that some items may be more sensitive to contextual or interpretation factors (46). Nevertheless, the minor deviations found in this study are relatively common and can likely be attributed to the linguistic and developmental characteristics typical of younger populations (47).

Analysis of the correlations between each item and the total SMOSS-C score produced positive results, which are consistent with those of the other reliability tests and previous validations of the instrument (13, 18). All the items showed significant correlations with the total questionnaire score (*p* < .01), supporting the idea that each item contributes significantly to measuring the overall construct. The Spearman's *ρ* coefficients ranged from 0.337 to 0.777, indicating a moderate to strong relationship between the individual items and the overall score. However, item 15 was identified once again as having the lowest correlation with the total score (Spearman's *ρ* = .337). This suggests that this item may measure aspects that generate ambiguous interpretations by children (43). This observation coincides with the issues identified in internal consistency and ICC analyses, emphasizing the need for revision.

A critical strength of this study was the inclusion of McDonald's omega (ω) alongside Cronbach's alpha (α). By reporting omega values of .892 (test) and .886 (retest), we addressed the inherent limitations of alpha regarding the assumption of tau-equivalence (a constraint frequently violated in developmental research where item-factor loadings can vary). These high coefficients indicate that the items represent the underlying construct consistently, ensuring that the total scores are reliable for assessing the social motivational orientations in sport in children. Furthermore, the stability of these coefficients across the two measurement points, coupled with the Bland-Altman plot analysis, provides a rigorous validation of the scale's reliability. Unlike traditional correlation coefficients, the Bland-Altman approach confirmed a mean bias near zero with tight limits of agreement, demonstrating that the SMOSS-C is not significantly affected by systematic measurement errors or learning effects during retesting. While some items exhibited ceiling effects (particularly Item 15) the overall high comprehensibility scores across all age groups (6–12 years) suggest that the instrument is conceptually and linguistically appropriate for its target population. The combination of high internal consistency and strong temporal agreement positions the SMOSS-C as a reliable tool for both clinical and educational settings.

One of the main strengths of the SMOSS-C is its rigorous linguistic and cultural adaptation process. This approach involves direct and reverse translation in accordance with international recommendations, as well as adapting the content to suit the cognitive, linguistic, and socioemotional levels of children aged 6–12. Another notable strength of the SMOSS-C is its applicability in real-life educational and sporting contexts. Primary education plays a crucial role in developing attitudes, values and motivations toward PA. Having a valid and reliable tool enables the diagnosis of motivational profiles and the guidance of more effective pedagogical interventions adapted to students' needs. In this sense, the SMOSS-C can be used by PE teachers, coaches, school counsellors, and sports professionals to (1) identify the social factors that influence children's motivation to play sports (e.g., group acceptance, the desire for recognition and social pressure); (2) design motivational strategies that are tailored to the profile of their students or athletes; and (3) evaluate the impact of intervention programs on social motivation in school or extracurricular sporting contexts. The brevity and accessible format of the questionnaire make it a practical tool for use with young children, as it minimizes their risk of becoming fatigued or losing attention while completing it.

Importantly, this study aligns with a recent article on the development of the first assessment model for physical literacy in Spain: the Spanish Physical Literacy Assessment for Children (SPLA-C) (48). In this Delphi study, national experts concluded that this scale should form part of the 'social interaction and barriers to physical activity practice' component of the new PL assessment model in Spain. As part of the SPLA-C model, the SMOSS-C enables schools and policymakers to monitor students' social development in relation to the PL. This information can be used to design more holistic PE programs that address not only physical skills but also students' social motivation to participate in sports.

While the results obtained support the validity and reliability of the SMOSS-C for use with children, it is important to recognize the following limitations, which should be considered when interpreting the findings and guiding future research.

First, possible differences in the instrument's structure or reliability according to gender, level of sporting experience or specific age were not explored. A limitation of the present study is that subgroup analyses examining measurement equivalence or invariance across gender, age, and sport experience levels were not conducted. Such analyses are important, particularly given children's age-dependent comprehension, which could influence item interpretation and scale performance. Future research will aim to perform configural, metric, and scalar invariance tests, especially across gender and age groups, to ensure the instrument functions equivalently across subpopulations. Given the current sample size (*n* = 128), we acknowledge that statistical power may have been insufficient to support these analyses, which should be addressed in larger-scale studies.

Second, the use of a self-reported format poses an inherent challenge when working with young children, given that their ability to self-reflect or interpret abstract statements can vary considerably. Although cognitive interviews were conducted in the pilot phase, comprehension problems were evident in some items (such as items 6 and 15), suggesting that, while the linguistic adaptation was generally adequate, a more focused review of these elements could be beneficial. Therefore, future studies should conduct a more in-depth qualitative analysis of problematic items through interviews or focus groups with children, with the aim of improving their wording and comprehension. Moreover, children's responses may be influenced by social desirability, which we did not control for in this study. Similarly, future research could involve developing illustrated versions or versions supported by visual elements to facilitate use with younger children or those with reading comprehension difficulties. Also, Future studies could apply IRT/Rasch analyses to examine item difficulty and discrimination across age levels.

Finally, the cross-sectional nature of the study is a limitation, inviting future longitudinal studies to explore the sensitivity of the SMOSS-C to educational interventions or motivation programs and evaluate its ability to detect significant changes in motivational orientations over time.

This study made it possible to linguistically and culturally adapt the SMOSS for use with Spanish children aged 6–12 years. The resulting children's version is known as the SMOSS-C. Analyses show that the adapted version has a valid factor structure, as well as adequate levels of internal consistency and temporal reliability for most items and the overall questionnaire score. Although two items (i.6 and i.15) were identified as being less reliable and more difficult to understand, the overall body of psychometric evidence supports the use of the SMOSS-C as a reliable and useful tool for assessing social motivational orientations in sports among school-aged children. Using the SMOSS-C can improve our understanding of the social factors influencing children's motivation for PA and help us design more effective pedagogical strategies in education and sport. The SMOSS-C is an important step toward providing assessment tools that are adapted to the reality and needs of children's educational contexts. It promotes more motivating, inclusive, and meaningful physical and sporting practices from an early stage of development. The SMOSS-C is part of the social domain of the SPLA-C, the first model for the assessment of PL in Spain.

## Data Availability

The raw data supporting the conclusions of this article will be made available by the authors, without undue reservation.
