# Supplementary material for: Spanish translation and cross-cultural adaptation and validation of the social motivational orientations in sport scale for children
Source: Front Sports Act Living. 2026 Feb 4;8:1743553. doi: 10.3389/fspor.2026.1743553 (PMC12914941; doi:10.3389/fspor.2026.1743553)
Supplement: Supplementary file 1 [file Table1.docx]

Supplementary Material

# Supplementary Data

**SOCIAL MOTIVATIONAL ORIENTATIONS IN SPORT SCALE FOR CHILDREN (SMOSS-C).**

**SPANISH VERSION**

**Código:**

*Totalmente de acuerdo*

*Totalmente en desacuerdo*

Me siento bien en mi deporte cuando…

| 1. Los/las demás me dicen que lo he hecho bien. | 1 | 2 | 3 | 4 | 5 |
| --- | --- | --- | --- | --- | --- |
| 2. Hago buenos amigos/as en el equipo. | 1 | 2 | 3 | 4 | 5 |
| 3. Soy parte de los populares del equipo. | 1 | 2 | 3 | 4 | 5 |
| 4. Mis compañeros/as de equipo y yo nos reímos juntos. | 1 | 2 | 3 | 4 | 5 |
| 5. Soy el centro de atención. | 1 | 2 | 3 | 4 | 5 |
| 6. Hago nuevos amigos/as con los que me junto fuera del deporte. | 1 | 2 | 3 | 4 | 5 |
| 7. Me divierto con los/las demás de mi equipo. | 1 | 2 | 3 | 4 | 5 |
| 8. Soy parte de los/las niños/as populares. | 1 | 2 | 3 | 4 | 5 |
| 9. Los/las demás niños/as piensan que soy muy bueno/a en el deporte. | 1 | 2 | 3 | 4 | 5 |
| 10. Los/las demás me felicitan por mis logros. | 1 | 2 | 3 | 4 | 5 |
| 11. Disfruto pasando el tiempo con el resto de los/las jugadores/as. | 1 | 2 | 3 | 4 | 5 |
| 12. Me hago amigo/a de otros/as niños/as que practican mi deporte. | 1 | 2 | 3 | 4 | 5 |
| 13. A los/las demás les impresiona lo bien que juego. | 1 | 2 | 3 | 4 | 5 |
| 14. Soy uno de los/las jugadores/as más populares. | 1 | 2 | 3 | 4 | 5 |
| 15. Me divierto pasando el rato con los/las demás | 1 | 2 | 3 | 4 | 5 |
